# Stress in School. Some Empirical Hints on the Circadian Cortisol Rhythm of Children in Outdoor and Indoor Classes

**DOI:** 10.3390/ijerph14050475

**Published:** 2017-04-30

**Authors:** Ulrich Dettweiler, Christoph Becker, Bjørn H. Auestad, Perikles Simon, Peter Kirsch

**Affiliations:** 1 Department of Cultural Studies and Languages, University of Stavanger, 4036 Stavanger, Norway; 2 Department of Sports and Health Sciences, Technical University of Munich, Arcisstr. 21, 80333 Munich, Germany; chris.becker@tum.de; 3 Department of Mathematics and Natural Sciences, University of Stavanger, 4036 Stavanger, Norway; bjorn.auestad@uis.no; 4 Faculty of Social Science, Media and Sport, Johannes Gutenberg University, Mainz Saarstr. 21, 55099 Mainz, Germany; simonpe@uni-mainz.de; 5 Department of Clinical Psychology, Central Institute of Mental Health, Medical Faculty Mannheim, University of Heidelberg, J 5, 68159 Mannheim, Germany; Peter.Kirsch@zi-mannheim.de

**Keywords:** stress, cortisol, physical activity, outdoor learning, mixed effect model

## Abstract

This prospective longitudinal survey compared the stress levels of students taught using an outdoor curriculum in a forest, with children in a normal school setting. We were especially interested in the effect outdoor teaching might have on the children’s normal diurnal cortisol rhythm. 48 children (mean age = 11.23; standard deviation (SD) = 0.46) were enrolled, with 37 in the intervention group (IG), and 11 in the control group (CG). The intervention consisted of one full school day per week in the forest over the school year. Stress levels were measured in cortisol with three samples of saliva per day. Furthermore, the data allowed for statistical control of physical activity (PA) values. For data analysis, we used a linear mixed-effects model (LMM) with random intercept and general correlation matrix for the within-unit residuals. The LMM yields that IG have expected greater decline of cortisol compared to CG; rate 0.069 µg/L vs. 0.0102 µg/L (log-units/2 h), *p* = 0.009. PA does not show a statistically significant interaction with cortisol (*p* = 0.857), despite being higher in the intervention group (*p* < 0.001). The main effect in our measures was that the IG had a steady decline of cortisol during the school day. This is in accordance with a healthy child’s diurnal rhythm, with a significant decline of cortisol from morning to noon. This effect is constant over the school year. The CG does not show this decline during either measurement day. Further research is needed to fully explain this interesting phenomenon.

## 1. Introduction

Both public debate and epidemiological research show evidence of an increase of stress symptoms and stress-associated diseases over the past decade. This has been identified on an international level [1], but it is also found in Germany specifically [2]. According to a recent statement issued by German health insurance companies [3], 16.2% of all employee sickness-related absences are attributable to mental health disorders, with many of them associated to stress. This is an extreme increase, since only 2% of paid sick leave was attributed to mental health disorders about 40 years ago. The discussion about stress has also reached the school context, at least after a significant school reform in Germany, which reduced high-school duration by one year with almost the same curriculum [4,5]. 

From a developmental neurobiology perspective, childhood and adolescence can be described as very vulnerable phases in which biological systems develop. Stress experience during this age can influence an individual’s response to stressful events for their lifetime, mainly via the effects of an increased activation of the hypothalamic-pituitary-adrenal (HPA) axis, the main biological stress system, on the brain [6]. Stress exposure during childhood might therefore lead to a biologically-based susceptibility to stress-related illnesses later in life [7]. This is also related to lower academic achievement [8]. Measures to reduce stress and to build up stress resilience in schools need to be found. 

There is promising research that exposure to green environments has some positive effects on mental health. Green environments can be described as areas with a certain amount of non-built spaces, for instance, public parks, lakes, rivers or forests. ‘Green’ does therefore not necessarily mean ‘green’ as a color, but rather stands as a synonym for nature with all its different shapes. In their recent systematic literature review, James et al. found that neighborhood greenness, or vegetation, may affect health behaviors and outcomes, and increased physical activity and social contacts may result in decreasing stress [9]. Their findings accord with a previous systematic literature review by Lee and Maheswaran, who concluded that most studies reported findings that generally supported the view of green environments having a beneficial health effect. However, they found that many studies were limited by poor study design, failure to exclude confounding, bias or reverse causality, and weak statistical associations [10]. On a general level, Roe et al. could associate more green space in deprived urban neighborhoods in Scotland to lower levels of perceived stress and improved physiological stress. This was measured by diurnal secretion patterns of the stress hormone cortisol and a steeper (healthier) diurnal cortisol decline with 104 subjects [11]. A similar correlation of green space and mental health factors seems to hold also for short-term visits of green space. Aspinal et al. found a relationship between green environment, behavior settings and emotions. They investigated the emotional experience of a group of walkers in three types of urban environments, including a green space setting, using mobile electroencephalography (EEG) as a method to record and analyze the *n* = 12 subjects’ emotional experience. Their findings showed evidence when moving into the green space zone of lower frustration, engagement, arousal, and higher meditation; respondents showed higher engagement when moving out of it [12]. Certainly in accordance with these findings, Brantman and colleagues investigated the effect of a 90 min walk in a natural environment and found a reduction of blood flow in the subgenual anterior cingulate cortex, a region associated with stress regulation, as well as a reduction of rumination, a cognitive style associated with depression [13]. Interestingly, a lower activation in this brain region during acute social stress was found in individuals who grew up in a rural environment, compared to those who grew up in an urban environment [14]. A systematic literature review [15] compared effects of physical activity in outdoor natural environments with indoor environments on physical and psychological wellbeing. In contrast to being physically active indoors, physical activity in outdoor natural environments—so called green exercise—is associated with a decrease in tension, anger and depression. However, the authors conclude that the methodological quality of evaluated studies is poor and more high quality large-scale studies are needed in this field of research. On this basis, Rogerson, et al. [16] tested 331 participants before and after a 5 km run in four different natural environments, applying appropriate measurements and statistical analyses. Participants’ stress and mood improved from pre- to post-run, independent of the specific green environment. The authors concluded that exercise in green environments offers possible benefits to psychological wellbeing. 

In a series of studies, van den Berg and her team describe green space as a buffer between stressful life events and health for *n* = 4529 Dutch respondents [17]. They confirm the hypothesis that more time spent in green space is associated with higher scores on mental health and vitality scales, independent of cultural and climatic contexts, by comparing *n* = 3748 observations from four European cities [18]. They also describe gardening as an effective measure to promote neuroendocrine and affective restoration from stress in *n* = 30 active private gardeners [19].

A systematic literature review on the effects of school-based outdoor education programs on students’ health, physical activity, social and learning dimensions, however, revealed that only very limited research has been reported so far [20]. Of more than 7800 articles analyzed, only 13 have met the inclusion criteria, of which six are so-called qualitative case studies, and seven apply (mostly poor) quantitative methodology. All studies are consistent in describing at least some positive effects of outdoor teaching to the various variables, health effects, physical activity, social and learning behavior. With respect to stress and mental health in the outdoor teaching context, we could identify only one slightly relevant study in the literature: Gustafsson and his colleagues describe that mental problems decreased in boys, but not in girls, in an outdoor teaching setting when compared to a control condition without outdoor teaching. These statistically significant effects were observed for a “difficulties total score”, as well as for “emotional symptoms”, “conduct problems”, and “hyperactivity”. Data were collected with a parent-report questionnaire [21]. Additionally, a promising quasi-experimental study design of the impacts of education outside the classroom on students’ physical activity, well-being, and learning has been recently published. This study with *n* = 834 observations of children aged 9–13 years had been performed in Denmark in the past years, and the results are due in late 2017 [22]. Those results may be able to close some of the above-mentioned gaps in recent research and theory construction in “green exercise”.

Taken together, the studies mentioned above give some evidence for a protective effect of a natural environment or outdoor setting on biological stress systems that might be related to mental health, also in the school context. However, to our knowledge, there is no prospective control group study investigating the effect of outdoor teaching on biological measures of stress. Therefore, we conducted the present pilot study, in which we hypothesized that regular intervals of outdoor teaching over the course of one school year will have a stress protective effect and, accordingly, will result in less activation of the HPA-axis, as reflected by a steeper decrease of cortisol secretion over the school day. The normal diurnal cortisol rhythm displays high cortisol values directly after getting up, with a steady decrease over the day [23,24,25]. However, it has been reported that adolescents with a high score on the Children’s Depression Inventory show a reduced decline of morning cortisol, when compared to low scoring children [25]. Thus, cortisol appears to be a fitting measure for stress, with respect to mental health.

In addition, using an explorative approach, we investigated children’s physical activity (PA) levels. Previous research indicates that children’s PA-levels are consistently higher in outdoor education settings using natural environments, compared to normal indoor settings [26,27]. Since it is known that high PA can lead to higher cortisol levels [28], we needed to control whether the expected PA differences between outdoor and indoor would modulate potential differences in the stress response. 

## 2. Materials and Methods 

### 2.1. Participants, Intervention, and Data Collection

Participants were recruited from 5th grade students from a secondary school (German “Gymnasium”) in Heidelberg, Germany. At the time of the study planning, two classes participated in the outdoor teaching program, consisting of one compulsory school day per week in the forest with the regular curriculum, while two classes were run as a traditional class without outdoor teaching. Students from those classes were to serve as control group. However, due to parent demand, the school decided to offer the outdoor teaching to three classes just before the school year, reducing the available number of control participants by 50%. Therefore, we included five students from a 6th grade indoor teaching class into the control group (CG). Finally, we were able to include 48 students into the study, 37 in the IG, and 11 in the CG. However, although we had no drop-outs, due to students being absent during the school year, we were not able to sample data from all students at all time points. Table 1 summarizes the enrolment data. 

Regarding the sociodemographic and anthropometric variables, slight differences exist between groups for age, weight and height. This is explained by the inclusion of students from 6th grade in the control group. There is, however, no statistically significant difference between the groups with respect to those variables that could potentially influence the biological measures. Nor was there a statistically significant difference in gender distribution (Table 2). The socio-economic status can be considered similar. 

The intervention consisted of one school-day per week in the forest. Thus, the overall mental load for the children is systematically the same, however, differently organized in the intervention. Looking at the respective schedules (Table A1 in Appendix A), two major differences can be seen: (1) the curriculum in IG is taught in cross-disciplinary units on the forest days, whereas it is taught in segments, subject by subject, in the CG; and (2) the pedagogical approach of the outdoor-learning program includes opportunities to be physically active on students’ free choice, as well as planned walks to reach specific places in the forest. Due to traditional indoor teaching concepts, such opportunities are rather limited for students within CG. According to the judgement of the school’s headmaster and teachers, the mental load of the five included sixth-graders in the CG can be compared to that of the other students in the CG. However, their curriculum is different, while the daily routines are the same.

Examination of the stress reactivity was performed by means of salivary cortisol analyzes with samples taken at three time points (8:30 AM, 10:30 AM, 12:30 PM) over the school year (seasons “fall”, “spring”, “summer”). Salivary cortisol was determined at the Biopsychology Laboratory at the Technical University Dresden, using a commercially available luminescence immunoassay (IBL, Hamburg, Germany). Due to the high variability of individual cortisol levels, the base-line is defined by the individual morning cortisol values at 8.30 AM, since we were only interested in relative individual cortisol concentrations over the day.

Physical activity of the control and intervention groups was determined by means of acceleration sensors. For this purpose, one Axivity AX3 sensor (Axivity Ltd., Newcastle upon Tyne, UK) was attached to each child’s back above the upper point of the posterior iliac crest, with the aid of medical tape. Moderate-to-vigorous physical activity (MVPA) is a reference criterion for determining the recommended physical activity in children and adolescents [29]. As a first step, we converted the raw vector magnitude acceleration data to Actilife-format via an in-house software developed by the University of Southern Denmark. Afterwards, the children’s MVPA was analyzed in Actilife v.6.11. 4 (ActiGraph, Pensacola, FL, USA). Cut-points reported by Romanzini et al. [30] have shown a good validity among children and adolescents at every activity level and were used to identify MVPA.

In order to determine long-term effects of the intervention on stress levels and to cross-validate the saliva measures, hair probes had been taken for ex-post analyzes of cortisol levels following the measurement days. However, data from the hair samples are not included in the present paper because of missing data, mostly due to hairs being too short to receive reliable probes. 

### 2.2. Data Analyses

To account for the repeated measurements structure of the data and the complexity of interactions, we fitted linear mixed-effects models (LMMs), using the software package nlme [31,32] in R 3.3.2 (31 October 2016) [33], and JASP [34]. Without interaction terms, the general model for our analyses at time point j and season *k* = “fall”, “spring”, “summer” for individual *i* is:

*Y_ijk_* = β_0_ + *b_i_* + β_1_(*season*, *k*) + β_2_(*time.point*, *j*) + β_3_(*group*) + ε*_ijk_*, (1)

where β_0_ is the intercept and the *b_i_*’s are the random intercepts being independent zero mean normally distributed random variables. The residuals ε*_ijk_* are also zero mean normally distributed random variables with covariance matrix dependent on the situation as described below. Group is an indicator variable showing whether individual i belongs to the intervention or control group. The full factorial model, i.e., including all up to third order interactions between the fixed factors, was checked as a starting point.

With respect to the activity data, we analyzed the time points from 8:30 AM to 10:30 AM (“midmorning”), and from 10:30 AM to 12:30 PM (“noon”), then comparing those sets of moderate-to-vigorous physical activity (MVPA) over the fixed effects. We compared this model to less complex models by removing interactions, and had to include within-individual heteroscedasticity as a weighing factor (power of variance covariate) in order to include adjustment for residual variance dependent on MVPA values [35,36]. The model fit was evaluated using the AIC criterion and likelihood ratio tests (cf. Table S1). The model without 3rd order interaction showed the best fit to the data (cf. Figure S1).

With respect to the cortisol data, we had to include a general correlation matrix for the within-unit residuals. This resulted in a clearly better fit compared to using independent residuals. In order to obtain more symmetric data distributions facilitating assumption of normality, the cortisol values were log transformed. Starting from a full factorial model including the third order interaction between group, time, and season, the third order interaction, and the interaction between time and season, were excluded according to the Akaike information criterion (AIC), which is a measure of the relative quality of statistical models for a given set of data, and likelihood ratio tests. The resulting model showed good fit to the data according to residual plots (cf. Figure S2).

Since physical activity can result in higher cortisol values, we controlled cortisol against physical activity [28]. We therefore set up another set of models to analyze the interactions of MVPA- and cortisol-measures. In a first approach, we tested the accumulated MVPA-values from the subsets 8:30 AM–10:30 AM, and 10:30 AM–12:30 PM, respectively, against the difference of cortisol measures (diff_logCortisol) at 10:30 AM compared to 8:30 AM, and 12:30 PM compared to 10:30 AM, respectively, using a linear mixed effects model with random intercept (2).

*Y_ijk_* = β_0_ + *b_i_* + β_1_(*MVPA*) + β_2_(*season*, *k*) + β_3_(*time.point*, *j*) + β_4_(*group*) + ε*_ijk_*, (2)

*Y_ik_* = β_0_ + *b_i_* + β_1_(*Sum_MVPA*) + β_2_(*season*, *k*) + β_3_(*group*) + ε*_ik_,* (3)

Again, we compared the full interaction models to less complex models by removing interactions. The model fit was evaluated using the AIC criterion and likelihood ratio tests. The model without the interaction over time showed the best fit to the data. Additionally, we tested the full accumulated MVPA-values at 12:30 PM (Sum_MVPA) against the overall difference of the cortisol values (delta_logCortisol) in another set of models (3) with varying interactions, including random intercept effects. The inclusion of a general correlation matrix for the within residuals in the model was not necessary according to the AIC criterion (cf. Table S3, Figure S3). Again, the model without interaction over time (season) showed better fit to the data than the full model.

## 3. Results

### 3.1. Physical Activity

**Figure 1:**
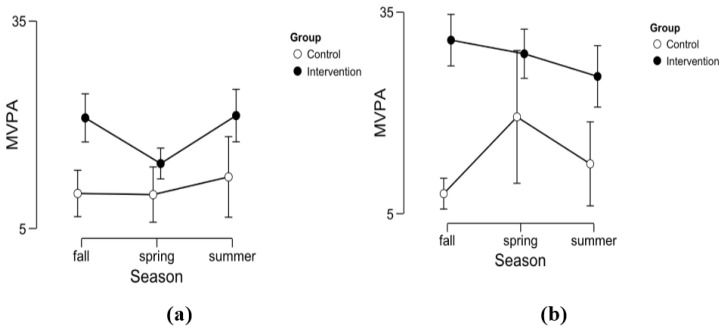
Here, the different moderate-to-vigorous physical activity (MVPA) levels are displayed for (**a**) the accumulated values from 8:30 AM to 10:30 AM, and (**b**), the accumulated values from 10:30 AM to 12:30 PM with respect to seasons and group. The descriptive parameters can be seen in Table 3. The error bars indicate the 95% confidence interval (CI). Inferential analyses reveal that intervention group (IG) are estimated 11:30 min longer in MVPA levels (SE = 2.08) than the control group (CG) (*p* < 0.001) per 2-h time interval (cf. Table A3 in the Appendix). The difference is especially bigger in the second half of the school day (*p* < 0.001).

As could be expected with respect to the school setting, the children in the outdoor classes show higher activity levels than their peers in the school building. Table 3 gives detailed descriptive information on the data, and Figure 1 displays a graphical output.

The main effect revealed by the linear mixed effect model is as follows: children in the forest group are expectedly 11:30 min longer in MVPA-level per 2-h-unit than their peers back in school (*p* < 0.001), averaged over the whole school year. The difference is especially larger in the second half of the school day (difference: 7:54 min; *p* < 0.001) (cf. Table A3 in Appendix C). Seasonal effects can also be observed. However, the expected time in MVPA in spring and summer is relatively shorter for IG than CG (difference spring: −7:36 min, *p* < 0.000; difference summer: −6:30 min, *p* = 0.004), which is due to a light decrease of time spent in MVPA in IG over the seasons with its lowest value in spring. Meanwhile, time spent in MVPA simultaneously increases in the CG with its highest value in spring. Comparing the means of the accumulated time in MVPA over the three measurement days without accounting for seasonal or diurnal differences shows that IG spent more than twice as much time in MVPA than CG (M_IG_ = 47.18 min, M_CG_ = 23.28 min; *t* (51.162) = −7.763, *p* < 0.001).

### 3.2. Cortisol Measures

**Figure 2:**
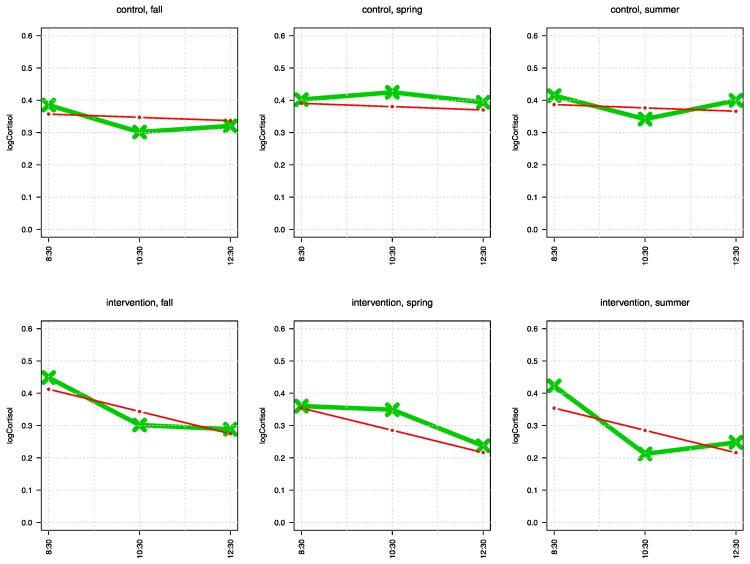
Displayed are the running curves of logCortisol over the day in each season for both groups. The upper panels show the CG values, the lower panels IG values. The green line represents the mean values, the red line connects the calculated values of least squares regression. It can be seen that IG shows, in contrast to CG, a clear decrease of cortisol levels in the course of the school days, but not the CG (*p* = 0.009).

The cortisol measures are log-distributed, as can be seen from the Table A2, and the graphical displays in Appendix B. Thus, we performed the statistical analyses of cortisol with logarithmized data. The linear mixed effect model of the log-cortisol data yields that the intervention group in the outdoors have a statistically significant greater decline of cortisol compared to the control group; rate 0.0102 µg/L + 0.0588 µg/L = 0.069 µg/L vs. 0.0102 µg/L (log-units/2 h, *p* = 0.009) (cf. Figure 2). Moreover, the intervention group has expected lower cortisol levels in spring at the half-year compared to control group, difference: 0.0915 µg/L, *p* = 0.050, which is still statistically significant for the end of the school year, difference: 0.0879 µg/L, *p* = 0.052 (cf. Table A4 in Appendix C). 

### 3.3. Interaction of Cortisol Measures and Physical Activity

Both strategies of testing for interaction between the cortisol measures and moderate to vigorous physical activity did not yield statistically significant results. Table A5 and Table A6 in Appendix C show the results of the above-mentioned interaction models (2) and (3).

## 4. Discussion

### 4.1. General Observations

The present study was conducted to investigate whether regular engagement in an outdoor teaching has a positive effect on stress responses in students, and whether this effect is associated with physical activity in this setting. 

The findings of the cortisol measures allow for a straightforward interpretation: In our case, teaching in the forest was associated with a lower cortisol secretion at noon, compared to the control group. Given that the normal diurnal cortisol rhythm displays high cortisol values directly after waking up, which steadily decreases over the day, the lack of such a decrease of cortisol over the school-day in the control group might be regarded as detrimental [23,24,25]. It has been shown that adolescents with a high score on the Children’s Depression Inventory also show a reduced decline of morning cortisol, when compared to low scoring children [25]. It could therefore be argued that the cortisol profile observed in the indoor class is rather similar to profiles observed in individuals prone to develop a stress associated mental disorder, such as depression. However, it is important to remember that cortisol secretion is influenced not only by psychosocial stress, but also by a number of other conditions including physical activity, mental load, or different positive stressors [37]. However, while we can exclude differences in physical activity as a potential factor underlying group differences (see above), we cannot exclude differences in mental load or eustress between groups. Explaining the effect with eustress seems implausible, since the participants in the forest classes did very likely not experience less positive events, and less fun, than those in school. With respect to positive social events, literature suggests that students in outdoor classes experienced more positive encounters during the school day than those in the indoor class [38], which would counter the cortisol effect observed in our study. The mental load is difficult to estimate. In principle, both groups had the same curriculum, with the exception of the five six-graders. However, the individual school subjects had not been absolutely synchronized, nor had the lessons been delivered by the same teachers (cf. Table A1 in the Appendix). Moreover, literature suggests that students taking part both in short- and long-term outdoor teaching programs likely do not consider the outdoor teaching as “regular school lessons,” despite long working hours and a very advanced curriculum [38,39]. Furthermore, they show a higher degree of long-term knowledge retention [38] and emotional connectedness to the curriculum [40,41]. Thus, some of the effect might be attributed to differences in mental load, especially defined by the teaching context. 

As mentioned before, the cortisol data are very likely not confounded by the students’ physical activity, as the statistically non-significant interaction analyses show. This is interesting in two respects—the “dosage” of physical activity on a “typical” outdoor schooling day seems to be such that children are: (a) comparatively very physically active but without; and (b) are impacted on their biological stress system. Hill et al. [28] report that a statistical increase of cortisol can only be reached at an exercise intensity level of 80% of the VO_2_ max for 30 min, which reflects a substantially higher exercise load than the one achieved in our intervention group. After corrections for circadian factors, lower exercise loads than 80% of VO_2_ max for 30 min may actually result in a reduction of circulating cortisol [28]. However, VO_2_ max related thresholds are relative to the individuals’ levels of physical exercise capacity, whereas the accelerometer data in our study are absolute measures of the amount of PA. Both measurement variables are related but not directly comparable. Thus, we cannot explore this further with our data. On the other hand, the lack of interaction between MVPA and cortisol suggests that the observed stress buffering effect of the outdoor teaching setting can be attributed to the specific environment. The natural environment of the forest offers potential—so far unspecified in the educational context—influences on: perception, social aspects, experiences and, specifically, exposure to sunlight [42]. These aspects can be subsumed as a so-called “green effect”. There is also some evidence from the literature that such a “green effect” [43,44,45,46], together with some learning psychological aspects [39,47], might add to the stress reduction in the intervention group. 

With respect to the findings of the children’s activity levels in the outdoor vs. the indoor classes, our results directly confirm (in part) previous Danish research by Mygind [26,27]. As reported, the outdoor classes in our study led to statistically significant higher PA levels, compared to the indoor classes. In both Danish studies, the involved students participated in similar regular curriculum-based outdoor education projects. Students’ PA was objectively measured during outdoor and indoor learning and compared intra-individually. Students’ PA was statistically significant higher during outdoor learning days, compared to traditional indoor school days. We cannot, however, explain the seasonal effects with either the literature or data from our study. From a practical perspective, we would consider those differences as artefacts, and not relevant. However, further research with more measures over the year might support another conclusion.

Taken together, our findings give some preliminary support for the often assumed, but so far empirically unconfirmed, hypothesis that outdoor teaching over regular intervals is beneficial to children’s mental and physical health [48], which supports our main hypothesis. This is some of the first research into biological stress factors in the outdoor education context. The results are therefore unique, but have some limitations. 

### 4.2. Limitations

Since the hair cortisol probes could not be reliably analyzed, we were not able to check for mid- or long-term or buffer-effects of the stress reduction in the outdoors, hence stress-resilience. Moreover, three measurement days over one school year provide too coarse a resolution for fully understanding the phenomenon. However, more measurement days were not possible for logistical and school organizational reasons, and more measures would have inevitably led to even less enrolment, and probably more drop-outs. Clearly, more research is needed to understand patterns at a finer level of detail. More insights into students’ diurnal cortisol responses could also be realized by testing the IG on normal indoor school days.

Another critical point for the present study is the non- or “quasi”-randomization of the participants into the particular groups. While the group allocation was not done by the experimenter, it was determined by school policies and according to the parents’ (and children’s) preferences. This might in itself bear some bias which cannot be corrected with statistical methodology. For ethical reasons, we could not test the children for behavioral or mental health disorders. The parents in favor of outdoor teaching against “normal” schooling might have children yielding a certain cluster of psychological straits we are not aware of, and whose statistical prevalence has not yet been researched. 

## 5. Conclusions

The main result of our study is that the children in the forest class show a steady decline of cortisol during the school day which was not observable in the control group. This is in accordance with a healthy child’s diurnal rhythm, and its statistically significant decline of cortisol from morning to noon. This effect is constant over the school year. The children in the classroom setting did not show this effect on either measurement day. However, our data gave no empirical hints to explain that interesting phenomenon. Despite the mentioned limitations of the current study, the cortisol data are consistent and valid. Further, the data supports the conclusion that outdoor education had a positive effect on stress responses in children in our intervention group, in contrast to indoor teaching in the control group. These novel findings, interesting as they are, only represent a first step towards a deeper understanding of the “stress in school” phenomenon measured with biological parameters. Larger prospective studies are needed to confirm the results, and to potentially test for consequences of reduced stress exposure in outdoor setting, with respect to mental health in children and adolescents. 

## Figures and Tables

**Table 1:** Enrolment data.

	Participants Recruited	Fall	Spring	Summer
Total	48	46	45	46
Intervention	37	35	35	35
Control	11	11	10	11

**Table 2:** Participant anthropometric data.

	Intervention Group	Control Group	Statistics
Age in fall	11.1 years	11.6 years	*p* = 0.073
Weight in fall	35.12 kg	35.67 kg	*p* = 0.79
Height in fall	145.3 cm	148.0 cm	*p* = 0.21
Gender	23 (62%) male 14 (38%) female	7 (64%) male 4 (36%) female	*p* = 0.93

**Table 3:** Descriptives-Moderate-to-vigorous physical activity (MVPA) [min].

Group	Time.Point	Season	Mean	SD	*n*
Control	midmorning	fall	10.068	4.996	11
		spring	9.900	5.589	10
		summer	12.458	5.551	6
	noon	fall	7.977	3.414	11
		spring	19.400	13.808	10
		summer	12.417	5.953	6
Intervention	midmorning	fall	21.000	9.655	32
		spring	14.396	6.399	34
		summer	21.333	8.984	24
	noon	fall	30.828	10.640	32
		spring	28.794	10.491	34
		summer	25.427	10.832	24
SD: Standard Deviation.					

**Figure A1:**
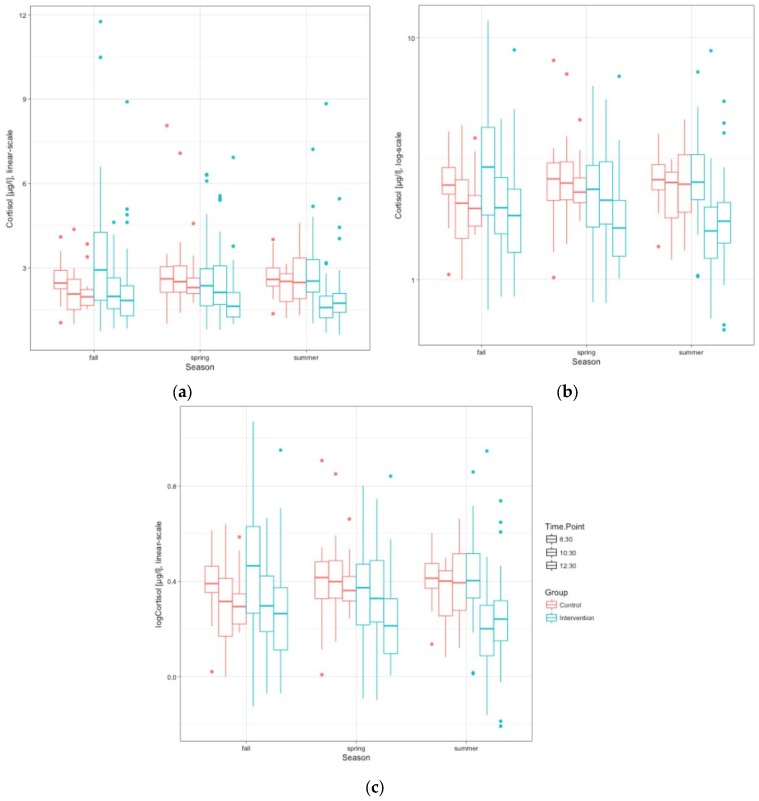
Cortisol raw values compared to log10-transformed cortisol values. Displayed are (**a**) the raw cortisol values on the three time-points (8:30, 10:30, 12:30) at the three seasons (fall, spring, summer) in both groups (control = red, intervention = green). Panel (**b**) shows the same data with a logarithmized y-axis and (**c**) the logarithmized cortisol-values on a linear y-scale. It can be seen that by log-transforming the y-scale (**b**), some outliers can be eschewed, and that by logarithmizing the cortisol-values (**c**), the variance, skewness, and kurtosis of the data can be reduced (cf. Table A1).

## Supplementary Materials

The following are available online at www.mdpi.com/1660-4601/14/5/475/s1, Figure S1: Standardized MVPA residuals, Table S1: Model fit parameters MVPA, Figure S2: Standardized logCortisol residuals, Table S2: Model-fit parameters logCortisol, Figure S3: Standardized logCortisol by MVPA interaction residuals, Table S3: Model-fit parameters logCortisol by MVPA interaction.

## Appendix A

**Table A1:** School schedule and measurement procedures.

Schedule	Time Data Collection	IG	CG
07.55–08.40	8:30	Meeting at 8.00 and short walk to outdoor “classroom”; preparing for the day	regular class according to curriculum
08.45–09.30		forest class according to curriculum	regular class according to curriculum
09.30–09.45		break	break
09.45–10.30	10:30	continued forest class according to curriculum	regular class according to curriculum
10.35–11.20		continued forest class according to curriculum	regular class according to curriculum
11.20–11.35		break	break
11.35–12.20		continued forest class according to curriculum	regular class according to curriculum
12.25–01.05	12:30	continued forest class according to curriculum	regular class according to curriculum

## Appendix B

**Table A2:** Descriptives for logCortisol and Cortisol.

	Cortisol	LogCortisol
Mean	2.445	0.3322
Std. Error of Mean	0.07080	0.01072
Std. Deviation	1.419	0.2150
Variance	2.015	
Skewness	2.413	0.3124
Kurtosis	9.287	0.2787

## Appendix C

**Table A3:** Summary of interaction analyzes MVPA [min].

	Value	Std. Error	DF	*t*-Value	*p*-Value
(Intercept)	9.361	1.59	178	5.889	0.000
IG	11.539	2.08	46	5.547	0.000
S_spring_	−0.107	1.198	178	−0.089	0.929
S_summer_	4.664	1.962	178	2.377	0.019
t_noon_	−0.775	1.107	178	−0.7	0.485
IG:S_spring_	−7.606	1.703	178	−4.466	0.000
IG:S_summer_	−6.526	2.249	178	−2.901	0.004
IG:t_noon_	7.914	1.657	178	4.777	0.000
S_spring_:t_noon_	7.767	1.806	178	4.3	0.000
S_summer_:t_noon_	−1.261	2.137	178	−0.59	0.556

IG = intervention group, t = time point of the day (morning, midmorning, noon), S = season (fall, spring, summer).

**Table A4:** Summary of interaction analyzes logCortisol [µg/L].

	Value	Std. Error	DF	*t*-Value	*p*-Value
(Intercept)	0.3675	0.0567	349	6.4843	0.000
t	−0.0102	0.0196	349	−0.5206	0.603
IG	0.1141	0.065	45	1.7555	0.086
S_spring_	0.0332	0.04	349	0.8302	0.407
S_summer_	0.0293	0.0392	349	0.7482	0.455
t:IG	−0.0588	0.0225	349	−2.6175	0.009
IG:S_spring_	−0.0915	0.0459	349	−1.9948	0.050
IG:S_summer_	−0.0879	0.0451	349	−1.9476	0.052

IG = intervention group, t = time point of the day (morning, midmorning, noon), S = season (fall, spring, summer).

**Table A5:** Interaction of physical activity on cortisol (segmented 8:30–10:30, 10:30–12:30).

	Value	Std. Error	DF	*t*-Value	*p*-Value
(Intercept)	−0.010	0.064	177	−0.163	0.871
MVPA	0.001	0.004	177	−0.180	0.857
IG	−0.096	0.080	45	−1.192	0.240
MVPA:IG	0.002	0.005	177	0.437	0.662

IG = intervention group, MVPA = moderate-to-vigorous physical activity level, segmented.

**Table A6:** Interaction of physical activity on cortisol (whole day).

	Value	Std. Error	DF	*t*-Value	*p*-Value
(Intercept)	−0.043	0.082	65	−0.528	0.600
Sum_MVPA	0.001	0.003	65	0.250	0.803
IG	−0.201	0.112	45	−1.790	0.081
Sum_MVPA:IG	0.001	0.003	65	0.389	0.699

IG = intervention group, Sum_MVPA = moderate-to-vigorous physical activity level accumulated over the school day.
