# Supplementary material for: Stress in School. Some Empirical Hints on the Circadian Cortisol Rhythm of Children in Outdoor and Indoor Classes
Source: Int J Environ Res Public Health. 2017 Apr 30;14(5):475. doi: 10.3390/ijerph14050475 (PMC5451926; doi:10.3390/ijerph14050475)
Supplement: Supplementary file 1 [file ijerph-14-00475-s001.docx]

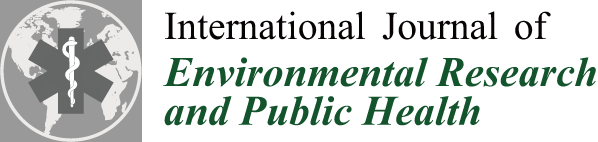
 *
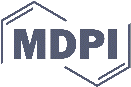
*

***Supplementary material***

**Table S1.** Model-fit parameters MVPA

| **Model** | **df** | **AIC** | **Test** | **p-value** |
| --- | --- | --- | --- | --- |
| lme.formula(fixed = MVPA ~ Group + Season + Time.Point + Group:Season + Group:Time.Point + Season:Time.Point, random = ~1 \| ID, weights = varPower()) | 13 | 1635.143 |  |  |
| lme.formula(fixed = MVPA ~ Group + Season + Time.Point + Group:Season + Group:Time.Point + Season:Time.Point, random = ~1 \| ID) | 12 | 1680.756 | 1 vs 2 | 0.000 |
| lme.formula(fixed = MVPA ~ Group + Season + Time.Point + Group:Time.Point + Season:Time.Point,  random = ~1 \| ID) | 10 | 1690.115 | 2 vs 3 | 0.001 |
| lme.formula(fixed = MVPA ~ Group + Season + Time.Point + Group:Season + Season:Time.Point,  random = ~1 \| ID) | 11 | 1688.846 | 3 vs 4 | 0.071 |
| lme.formula(fixed = MVPA ~ Group + Season + Time.Point + Group:Season + Group:Time.Point,  random = ~1 \| ID) | 10 | 1693.831 | 4 vs 5 | 0.008 |

**Figure S1. Standardized MVPA residuals**

**Figure S1.** This figure shows standardized residuals against fitted MVPA values allowing for within-group heteroscedasticity. We can assume good enough model fit.

**Table S2.** Model-fit parameters logCortisol.

| **Model** | **df** | **AIC** | **Test** | **p-value** |
| --- | --- | --- | --- | --- |
| lme.formula(fixed = logCortisol ~ Time.Point + Group + Season + Time.Point:Group + Group:Season, random = ~1 \| ID) | 10 | -155.401 |  |  |
| lme.formula(fixed = logCortisol ~ Time.Point + Group + Season + Time.Point:Group + Group:Season, random = ~1 \| ID, correlation = corSymm(form = ~1 \| ID)) | 46 | -164.955 | 1 vs 2 | < 0.001 |

**Figure S2. Standardized residuals logCortisol**

**Figure S2.** This figure shows standardized residuals against fitted logCortisol values. The distribution appears to be unbiased and homoscedastic. We can assume fairly good model fit.

**T**able S3: Model-fit parameters logCortisol by MVPA interaction.

| **Model** | **df** | **AIC** | **Test** | **p-value** |
| --- | --- | --- | --- | --- |
| lme.formula(fixed = delta_logCortisol ~ Sum_MVPA * Group, data = MVPA_diffCort, random = ~1 \| ID) | 6 | -30.974 |  |  |
| lme.formula(fixed = delta_logCortisol ~ Sum_MVPA * Group, data = MVPA_diffCort, random = ~1 \| ID, correlation = corSymm(form = ~1 \| ID) | 9 | -27.850 | 1 vs 2 | 0.411 |
| lme.formula(fixed = delta_logCortisol ~ Sum_MVPA * Group * Season, data = MVPA_diffCort, random = ~1 \| ID) | 14 | -26.537 | 2 vs 3 | 0.123 |
| lme.formula(fixed = delta_logCortisol ~ Sum_MVPA * Group * Season, data = MVPA_diffCort, random = ~1 \| ID, correlation = corSymm(form = ~1 \| ID) | 17 | -23.063 | 3 vs 4 | 0.471 |

**Figure S3: Standardized logCortisol by MVPA interaction residuals**

**Figure S3.** This figure shows standardized MVPA residuals against fitted logCortisol values over the whole school day without interaction in time. The distribution appears to be unbiased and homoscedastic. We can assume fairly good model fit
